# Influencing factors for effective teaching evaluation of massively open online courses in the COVID-19 epidemics: An exploratory study based on grounded theory

**DOI:** 10.3389/fpsyg.2022.964836

**Published:** 2022-08-05

**Authors:** Jingkuang Liu, Yanqing Yi, Xuetong Wang

**Affiliations:** ^1^School of Management, Guangzhou University, Guangzhou, China; ^2^Sciences of Civil, Environmental and Architecture Engineering, University of Padova, Padova, Italy

**Keywords:** MOOCs, effective teaching, influencing factors, grounded theory, semi-structured interview

## Abstract

Many factors affect the teaching of massively open online courses (MOOCs). In this study, to explore the factors that influence the effective teaching of MOOCs, a large number of relevant studies are analyzed. Based on grounded theory, semi-structured interviews were conducted with 30 students and teachers who used MOOCs for online teaching. The interview data were subjected to four research processes –open coding, axial coding, selective coding, and saturation testing– to explore the factors influencing MOOCs’ effective teaching and the interactions between them. The results demonstrate that: (1) Effective teachers, effective tuition, effective communication, active online learning, social support guarantees, and online course design have important positive effects on effective teaching, while only certain online learning behaviors will seriously affect the teaching effectiveness of MOOC, resulting in negative effects. (2) Effective communication is essential for effective teaching in MOOCs; effective teachers are the leading factor, thus teachers should take the initiative to study and understand the students to understand their various learning needs and difficulties. (3) Reasonable and effective classroom teaching design is key to improving MOOCs’ teaching efficiency. (4) E-learning is respected, cared for, and valued by society, including cognition, emotion, and learning platform support from family, school, teachers, and classmates, and has an important impact on students’ motivation and the effects of online learning. The results of this study further clarify factors influencing effective teaching of MOOCs, thus helping to enrich and supplement the theory of effective teaching and evaluation and providing theoretical guidance for teachers to effectively implement MOOC teaching.

## Introduction

At the start of 2020, the worldwide spread of COVID-19 caused extensive, unprecedented levels of disruption, with particularly pronounced impacts on normal classroom-based teaching (Huang and Lu, 2021). As a result of the pandemic, students were unable to attend school normally and normal online or offline teaching work could not be carried out. To ensure the health and safety of teachers and students and address the problem of education during the COVID-19 epidemic, the Chinese Ministry of Education issued the guidance of *Doing Well in the Organization and Management of Online Teaching in Colleges and Universities during the Epidemic Prevention and Control Period* on February 5, 2020; these guidelines specify the teaching policy of “online teaching and online learning”, within which massively open online courses (MOOCs) are one of the approaches used for crisis management. Under this framework, colleges and universities require students to take MOOCs to acquire new knowledge and ensure the continuity of their education (Ministry of Education of the People’s Republic of China [MOE of PRC], 2020). As a result, colleges and universities all over the country actively responded to the call of the Chinese Ministry of Education and implemented online teaching. College teachers actively accepted MOOCs teaching, which allowed high efficiency in practice to be achieved through ideological changes. In a short period before the beginning of the teaching term, university teachers extensively explored the use of MOOC teaching platforms and teaching tools (Duan, 2022). In this time, they quickly mastered functions including online clocking-in, course content upload, classroom discussion, testing, and data statistics, in addition to scanning, photographing, adding notes and uploading course material, replacing chalk blackboards with mouse pointers and touch screens, and facing students directly using webcams. Colleges and universities worldwide aimed to realize “continued teaching and learning with suspended classes” through MOOC teaching (Chang et al., 2019). Since 2012, MOOC teaching has gradually become more widely known and accepted by teachers and students in colleges and universities, which has helped to ensure the smooth transition to online approaches to teaching and students’ classroom learning during the COVID-19 epidemic (Zhou et al., 2020; Floss et al., 2021). However, due to the relatively short development time of MOOCs, their educational value remains difficult to measure. Furthermore, at present, there is no recognized systematic and universally effective teaching experience for application to MOOCs; there is also no evaluation index system to measure teaching in MOOCs, thus, these courses’ effectiveness is difficult to evaluate. In the United States, where MOOCs are developing rapidly, there are still numerous researchers who have questioned the educational value of these courses (Conole, 2016). Effective teaching is evaluated based on the students’ learning efficiency, including the effectiveness of the teaching process, which is characterized by the effectiveness and efficiency of the results of teaching rather than the teacher’s teaching content or the completion of teaching tasks (Devlin and Samarawickrema, 2010, 2022). However, due to the open nature of MOOCs, it is difficult to define teaching effectiveness in this course type. First, MOOCs, compared to traditional classroom teaching, have a very low average completion rate, but the learning completion rate alone cannot objectively reflect whether MOOC teaching is effective or not (Zhao et al., 2017). In addition, it is difficult to evaluate the effectiveness of MOOC teaching through qualitative methods such as observation, face-to-face communication and discussion, achievement display, evaluation, etc. While these methods can be applied to traditional classroom teaching situations, they are not feasible for MOOCs due to the large numbers of learners enrolled in the courses. To quantitatively evaluate the teaching effects of MOOCs based on big data technology, it is necessary to first define the evaluation dimension and key factors that influence the effectiveness of MOOC teaching and then explore and analyze which of these factors are the most important. In short-how do these key factors affect the effective teaching effect of MOOCs? On this basis, the following two objectives are set in this study:

(1) To explore the factors influencing the effective teaching of MOOCs in the context of an epidemic situation and what indicators can be used to measure effective teaching in MOOCs;

(2) To analyze the relationships between various influencing factors and the effective teaching of MOOCs and to evaluate the effective teaching of MOOCs by analyzing these factors.

To achieve these objectives, in this study, the grounded theory method is used to comprehensively and systematically review the representative literature related to evaluating effective teaching both locally and abroad. Through interviews with university students who have conducted MOOC teaching in various parts of the country, the factors influencing MOOC teaching in the context of the COVID-19 epidemic are summarized. This allows: (1) further analysis of the relationship between each measurement indicator and effective teaching, (2) formation of an effective teaching evaluation system of MOOCs, and (3) construction of an effective teaching evaluation influencing factor model. Finally, teaching behaviors in MOOCs that are beneficial to improving students’ learning performance are analyzed and explored to help future MOOCs effectively evaluate teaching, provide substantive suggestions for improving online teaching effectiveness, and further improve online teaching evaluation systems.

## Literature review

Pedagogy broadly refers to the teaching and learning activities of teachers and students, while effective teaching means that teachers can follow teaching rules, adopt appropriate teaching methods, successfully guide students to learn, and effectively achieve their expected teaching goals, allowing students to achieve academic progress or ability development to a certain extent after a period of study. Borich, a famous American pedagogy expert, proposed the concept of effective teaching in the book *Effective Teaching Methods* (Borich, 2016). The principle of effective teaching is universally applicable, and all teachers and educational administrators should consider what types of teaching are effective and how these can be achieved (Sofyan et al., 2021). Overall, the effective teaching method usually means that teachers follow the objective laws of teaching activities, use professional teaching behaviors, rationally design the teaching process, distribute their limited time, energy, and material resources evenly among their students, realize their teaching objectives and students’ personality cultivation and all-round development, and achieve as many effects from teaching as possible (Li and Liu, 2015; Zhang et al., 2019). These aspects are reflected in the effectiveness of the whole teaching process, namely, effective teaching organization, effective teaching objectives, effective interaction, effective curriculum content, effective learning results, etc. (Lowenthal and Hodges, 2015; Margaryan et al., 2015; Wang et al., 2018).

Effective teaching evaluation is an important way to improve MOOCs’ quality. With the increasing popularity of MOOCs, teaching effectiveness in this course type has become a hot topic, with increasingly many researchers investigating MOOCs (Lai et al., 2012; Xia et al., 2019). Pursel et al. (2016) introduced the contemporary situation, trends, and prospects of MOOCs in detail and proposed that MOOCs would change the future of education. In addition, numerous studies have investigated MOOCs not only in the field of education but also in the fields of management science and computer science (Calderon et al., 1996; Chen et al., 2022). Studies of MOOC learning have gradually deepened from initial theoretical research to practical exploration and measurement indicators related to the effects of MOOC teaching have also been discussed in many empirical studies (Porter and Brophy, 1988).

The teaching effectiveness of MOOCs is generally believed to directly relate to the recognition of online learners and the development of MOOCs’ teaching (Bangert, 2006; Huang et al., 2017), with the success of MOOC teaching thought to be largely based on the positive reputation of teachers (Margaryan et al., 2015). Although online and offline teaching have different focuses, some of the effectiveness evaluation indicators adopted in traditional offline teaching approaches are also applicable to MOOCs. From the perspective of students, educators’ teaching quality represents an important factor to evaluate the effectiveness of MOOC teaching, including factors such as deep professional knowledge, flexible methods, the use of auxiliary materials, research and understanding of students, a sense of responsibility, etc., all of which can be evaluation indicators for effective MOOC teachers (Wu and Shang, 2019; Zheng et al., 2021a,b). The learning effects for students can be measured through a series of indicators such as the learning experience and the learning results obtained by the course attendees after a period of participation (Zheng and Li, 2015; Mou, 2017). MOOC teaching is characterized by separation in both time and space, asynchronous feedback, and explicit teaching strategies, which differ significantly from traditional teaching in both teaching mode and method. Educators’ teaching styles and personal qualities will have a strong influence on the learning effect for students (De Moura et al., 2021; Zheng et al., 2021a,b). To some extent, teachers’ knowledge levels and mastery of teaching rules of online education influence the effectiveness of online teaching. Effective teaching includes clearly defined teaching objectives, real situational teaching content, catering to the needs of different students, clear explanations, examples, fair and just teaching evaluations, time-bound and timely feedback, and adjustment of teaching as required (Shen et al., 2020). To improve students’ satisfaction with online teaching, teachers can tailor their instruction to suit the characteristics of network teaching, adopt appropriate teaching transmission methods, stimulate students’ enthusiasm to learn, and create a positive classroom atmosphere. In the learning process of MOOCs, there are two-to-two interactive social network activities among students, teachers, network platforms, and courses (Devlin and Samarawickrema, 2010). Effective communication refers to the interactions between students and the curriculum, between students, and between teachers and students, i.e., the more interaction that occurs between teachers and students in the MOOC teaching process, the greater the learning enthusiasm of students and the more learning experience they will obtain, and the better the teaching effect will be (Jordan, 2014; Hew, 2016; Valdivia-Vázquez et al., 2018). Broadly, effective communication includes teacher–student teaching dialog, teacher–student cooperative learning, and network learning interaction. However, interactions between students are usually in the form of peer review, which can help to stimulate students’ learning motivation and turn external motivation into internal motivation. In comparison to traditional offline learning approaches, online learning systems are more open and student-centered, thus, the learning effect is largely determined by students’ online learning behaviors (Joo et al., 2018; Zhu et al., 2022). Therefore, students’ online learning behavior markedly affects the teaching effect of MOOCs. The online learning behavior variables of students enrolled in courses on MOOC platform websites that should be analyzed broadly include video learning behavior, homework learning behavior, forum learning behavior, learning time preference, page access, etc.

Self-study of teaching content represents one of the main learning methods for students in MOOCs, so it is very important for teachers to carefully design the course content and presentation. The design of MOOCs includes the course content, presentation mode, time flexibility of the course, the richness and practicality of the course’s content, the selection of teaching materials and the logic of their arrangement, the flexibility of class hours, and the diversity of content. Among these, flexible class hours and online virtual learning environments make the arrangement of class time more flexible and give students greater freedom, thus helping to address the communication challenges of traditional offline learning and instead improving students’ enthusiasm to listen to lectures (Alraimi et al., 2015). Online course design affects students’ interest in learning. Rich, interesting, and easy-to-understand course content will naturally attract students and stimulate their interest in learning, thus promoting the effectiveness of MOOC teaching (Zubkov, 2022a,b). In terms of the impact of the epidemic on MOOC teaching, Buttimer et al. (2022) believed that MOOC teaching reflected good fairness and security for black students. Yilmaz et al. (2021) believed that MOOC teaching could play a crucial role in disseminating accurate information to medical students in public health emergencies, and MOOC courses were well organized and effective.

However, to date, existing research generally only focuses on online-specific teaching effectiveness indicators or only analyzes evaluation indicators from traditional teaching; existing studies rarely combine effective teaching evaluation with MOOC teaching to formulate an evaluation system. In addition, previous studies rarely considered the impact of the epidemic on effective MOOC teaching, and the evaluation index system failed to reflect the impact factors of the epidemic. Thus, to evaluate the effectiveness of MOOC teaching, in this study, the effect of teaching MOOCs within an epidemic scenario is more comprehensively evaluated using the grounded theory method. In addition, the factors influencing MOOC classroom teaching effectiveness in undergraduate universities are explored, and an evaluation index system for effective MOOC teaching is constructed, including targeted suggestions to promote effective teaching.

## Approach and methodology

### Grounded theory

In this paper, interview results from university teachers and students using MOOCs were processed and analyzed by the grounded theory, which was proposed by socialists Glaser and Strauss in 1967 (Glaser and Strauss, 1967). This theory, which aims to explain and understand social phenomena, is a bottom-up inductive research process that advocates extracting relevant concepts from interview data to construct the theory, instead of deducing hypotheses from existing theories (Holt et al., 2022). The basic research logic of grounded theory involves collecting data through in-depth research on natural situations, comparing data with data continuously, summarizing concepts and categories from data through abstract and conceptual thinking and analysis, and building theories on this basis. The operating procedures of grounded theory generally comprise (Sun, 2011; Huang and Nie, 2021; Khan et al., 2022): (1) generating concepts from data and logging the data step by step; (2) constantly comparing data with concepts and systematically asking generative theoretical questions related to concepts; (3) developing theoretical concepts and establishing connections between them; (4) theoretical sampling, systematically coding the data; and (5) construction theory, which strives to achieve density, variation, and a high degree of integration of theoretical concepts. This study strictly follows the operational steps of grounded theory. Thus, concepts are first obtained from the original data, the concepts are then classified and summarized, the connections between concepts are evaluated again to obtain a concept network, and, finally, a theoretical framework is constructed.

### Sampling

In this study, students from key universities, general undergraduate universities, and some higher vocational colleges in China were interviewed between September 6 and December 30, 2020, through telephone and face-to-face interviews. A preliminary interview was conducted with several interviewees to further determine whether it was necessary to conduct in-depth interviews. If the conditions for the study were not met, the interview would be terminated immediately, and the interview materials would be deleted. In this study, a total of 30 college students were interviewed in depth, with the details of the interviewees shown in Table 1.

**TABLE 1:** Interview methods.

	Male	Female	Duration (min)
Online interview	10	6	30
Offline interview	7	7	35

Table 2 shows that 11 of the interviewees were from humanities and social sciences majors, 10 from polytechnic agronomy, seven from medical care and public health, and two from sports arts, indicating a wide range of sources, of whom 37.62% were freshmen, 36.38% were sophomores, and 25.7% were junior students (senior four students typically have very few or no courses to learn), indicating an even proportion, including 17 male participants and 13 female participants. In terms of the educational establishments involved, China’s “double first-class” universities (first-rate universities and disciplines), general undergraduate courses, and higher vocational colleges are all involved and representative, among which undergraduate colleges represent the majority (80.00%). Therefore, these data are considered sufficiently representative in terms of their overall characteristics and representativeness.

**TABLE 2:** Statistics of interviewees.

Background	Indicators	Number of samples	Proportion (%)
Gender	Male	17	56.67
	Female	13	43.33
Age	<18	2	6.67
	18–20	11	36.67
	21–23	13	43.33
	>23	4	13.33
Category of schools	Colleges or universities	24	80.00
	Junior colleges	6	20.00
Grade	Freshman	14	46.67
	Sophomore	10	33.33
	Junior	4	13.33
	Senior	2	6.67
Major	Humanities and social sciences	11	36.67
	Polytechnic agronomy	10	33.33
	Medical care and public health	7	23.33
	Sports and art	2	6.67

## Data analysis

According to existing references (Graham et al., 2001; Bangert, 2005; Brahimi and Sarirete, 2015; Hewawalpita et al., 2018; Jung and Lee, 2018; Deng et al., 2020; Liu et al., 2020; Reparaz et al., 2020; Blum-Smith et al., 2021; Li et al., 2021; Tang, 2021; Wu and Wu, 2021; Duan, 2022; Du and Qian, 2022), interview results, and a review of the teaching behaviors identified on MOOC platform websites (Such as: China University MOOC, Coursera, Udacity, and edX), the factors that influence effective teaching can be roughly divided into the following categories: (1) effective teachers, (2) effective teaching, (3) effective communication, (4) students’ online learning behavior, (5) social support guarantee, and (6) network course design. An effective teaching evaluation index system is constructed by these influencing factors. Step-by-step coding of interview and literature data forms the most important part of grounded theory. In this paper, open coding, axial coding, and selective coding were used to process the interview data, with the coding process carried out using Microsoft Excel 2013. This section describes the data analysis procedure in detail.

### Open coding

Open coding refers to assigning corresponding concepts to interviewees’ statements collected from interviews, thus forming a more general category. To exclude cases where individual special statements have no research meaning assigned to their categories alone, category assignment is only undertaken when more than one closely related concept for the statements was identified. Finally, a total of 23 categories was obtained, as shown in Table 3; for each category, only one representative statement is listed.

**TABLE 3:** Categorization of open coding.

Categories	Original statements (interview records)
Profound professional knowledge	A1: If teachers have profound and solid professional knowledge and a little knowledge of multidisciplinary and interdisciplinary professional knowledge, their teaching will be welcomed by more students.
Flexible methods	A2: Teachers can arrange courses by themselves, have various teaching methods, and are good at teaching students in accordance with their aptitude with the aid of auxiliary materials.
Knowing students well	A3: According to the different understanding and learning abilities of each student, teachers can understand these individual differences and set different learning requirements.
Responsibility	A4: Teachers are responsible for the teaching effects of students and take all classroom teaching seriously.
Clear and definite teaching objectives	A5: Teachers have clear teaching objectives for the course and will clearly explain to students the important and difficult points of the course content.
Real situational teaching content, taking care of different students	A6: I pay attention to personalized teaching. According to the differences of various students and the statistics of students’ achievement in achieving teaching objectives, and aiming at their differences in knowledge level, understanding ability, and application ability, I make use of the advantages of online teaching by setting different scenarios, demonstrating different examples, asking different questions, carrying out different inspirations, providing different methods, making different requirements, etc., so that students at different levels have the opportunity to complete teaching tasks, and effectively teach students in accordance with their aptitude, thus comprehensively improving the quality education of all students’ abilities.
Fair and just teaching evaluation	A7: Students can objectively and impartially evaluate and grade the teaching of the teaching teachers when carrying out the online teaching evaluation, including the fairness and impartiality of the evaluation process.
Timely feedback and adjustment of teaching	A8: Teachers can give timely feedback to students’ problems in the classroom, reflect on teaching through teaching feedback, and adjust the teaching plan as needed.
Teaching dialog between teachers and students	A9: Teachers can inspire students to think by asking questions, and students can also communicate with teachers on the network teaching platform. Through communication, not only students can get answers to questions and get the motivation to learn but also teachers can understand students’ current learning situation and stimulate students’ learning. Establishing an effective communication environment in network teaching activities can make teachers and students in different places communicate conveniently, thus effectively improving the quality of teaching activities and promoting effective communication between teachers and students.
Teacher–student cooperative learning	A10: Teachers can create a good classroom learning atmosphere for students and encourage cooperative learning between teachers and students.
Video learning	A12: When watching the video, students can interact with the teacher through QQ or other means so that they can ask the teacher immediately when they encounter any problems after watching the video.
Homework submission	A13: Regulations on the number of times students submit homework repeatedly: if errors are found during the closing time of the submission system, students can apply for resubmission, which can give students more time to think and reflect on the homework. The homework can be submitted in the form of PDF, pictures, or Word text format.
Forum learning	A14: When students use the course forum to communicate and learn, the browsing behavior of different gender learners in the course forum will be different. Female students are more active in browsing and posting in the course forum than male students. Outward students are the most active in posting in the course forum.
Learning time preference	A15: Teaching in the network environment reflects students’ dominant position, which is conducive to cultivating their interest, inspiring and inducing them to really mobilize their enthusiasm, initiative, and creativity in teaching. Some course videos can be watched back so that students can learn independently, control the pace and content of learning by themselves, leave some time and space for their thinking, and repeat learning of some important and difficult content to strengthen the learning effect.
Webpage access	A16: Students’ interest in this course can be judged by the number of visits to massively open online course (MOOC) pages, the number of people, and the time they stay on the pages.
Cognitive and emotional support from family, school, teachers, and classmates	A17: Families, schools, teachers, and classmates should support students’ learning in MOOCs. Parents should create a quiet external learning environment for students during online classes at home and allow them to frequently use electronic products due to online learning needs. At the same time, teachers can also understand the particularity of students’ learning environments at home and poor networks which lead to lost connections in class.
Support of learning platform	A18: Learning platforms launched by many famous universities in China, such as “xuetangX”, “UOOC”, and “CNMOOC”, have been launched on MOOC platforms one after another. Many famous universities in China, including Peking University, Fudan University, and Zhejiang University, have also built learning platforms on MOOC networks to provide more learning resources and space for learners.
Smooth network environment	A19: Smooth networks will have a certain influence on MOOC teaching, and stable wired or wireless networks can ensure that students can hear the complete course content taught by teachers.
Course content	A20: Teachers should be familiar with the course content in advance, prepare lesson plans for online courses, and present the content in pictures or videos according to the characteristics of online teaching, which is more vivid and easier to understand.
Mode of presentation	A21: Teachers’ teaching content can be presented in a combination of explanations, pictures, videos, and other ways.
Time flexibility of courses	A22: Network teaching is convenient, flexible, and fast. When students are unable to attend online courses due to special reasons, they can watch and download lecture videos, electronic lesson plans, courseware, and other teaching contents to make up for the deficiencies in the classroom, so it plays a role in deepening understanding, solving puzzles, and reviewing and improving.
The richness and practicability of the content	A23: At present, there is also an inter-school network platform, which can integrate the resources between teachers and schools and make the content of online courses richer and more practical.
Scientific selection of teaching materials and reasonable typesetting of materials	A24: Online teaching pays more attention to the selection of teaching materials and the typesetting of materials in teaching PPTs because the scientific selection of teaching materials and reasonable typesetting of materials can make the teaching content clearer and easier to understand.

### Axial coding

Spindle coding involves explaining the connotation of each category, further exploring and constructing the logical relationship between categories, classifying the initial categories, and then forming the main category. In this study, 24 categories were finally classified into seven main categories according to their logical relations and internal relations, as shown in Table 4.

**TABLE 4:** Main categories formed by principal axis coding.

Main categories	Corresponding categories	Connotation of categories
Effective teachers	A wide range of professional knowledge	Teachers’ extensive professional knowledge will increase students’ interest in the course.
	Flexible methods	Teachers use different methods to teach according to the contents of the class.
	Knowing students well	Teachers can understand the differences in students’ abilities.
	Responsibility	Teachers take every course seriously.
Effective teaching	Clear and definite teaching objectives	Teachers can accurately teach students the important and difficult points of the course.
	Real situational teaching content, taking care of different students	Teachers are good at contextualizing and visualizing the course content and teaching students in accordance with their aptitude.
	Clear explanation, examples	Teachers can clearly explain the content of the course and give examples in connection with specific events.
	Fair and just teaching evaluation	Students can objectively evaluate the teaching effects of teachers.
	Timely feedback and adjustment of teaching	Teachers can reflect on the students’ feedback and make timely adjustments.
Effective communication	Timely feedback and adjustment of teaching	Increasing interaction in the course can promote good communication between teachers and students, thus improving the teaching effect.
	Teacher–student cooperative learning	Teachers can create a good classroom atmosphere, and students also actively cooperate with teachers’ teaching, observe classroom discipline, and answer teachers’ questions.
Online learning	Video learning	Students can ask the teacher questions in time after class when watching teaching videos.
	Homework submission	Because they submit their homework through the network, students can have more time to think before submitting homework.
	Forum learning	Teachers and students can communicate and learn through forums.
	Learning time preference	The flexibility of online teaching time and space gives students more opportunities for autonomous learning.
	Webpage access	Students will have more views on the web pages of courses that they are interested in.
Social support guarantee	Cognitive and emotional support from family, school, teachers, and classmates	Parents and teachers can consider the instability of online teaching and understand the special situations of students.
	Support of learning platform	Learning platforms built by many famous universities using MOOCs provide more learning resources for teachers and students.
	Smooth network environment	A stable network environment can ensure that students can listen to the complete course content.
Online course design	Course content	The course content should be delivered to the students in a clear and understandable way.
	Mode of presentation	The presentation of teaching content can be a combination of explanations, pictures, and videos to enrich the content.
	Time flexibility of courses	What students do not understand in class can be reviewed repeatedly after class to deepen their understanding of difficult content.
	The richness and practicability of the content	Inter-school network platforms integrate teaching resources and increase the richness of online courses.
	Scientific selection of teaching materials and reasonable typesetting of materials	Reasonable PPT typesetting and material selection can make the course content more concise and easier to understand.

### Selective coding

Selective coding was used to further integrate and refine the six main categories in the aforementioned main axial coding table (Table 4). This approach was used to discover the core categories linked to the main categories, establish the correlation between them and other categories, and use the “storyline” method to connect the six main categories to form a relationship structure and, thus, construct a theoretical framework. The main category relation structure is shown in Table 5.

**TABLE 5:** Relationship structure of main categories.

Relational structure	Connotation of relational structure
Effective teachers → effective teaching evaluation	Extensive professional knowledge, flexible methods, use of auxiliary materials, research and understanding of students, and a sense of responsibility influence effective teaching evaluation.
Effective teaching → effective teaching evaluation	Clear and definite teaching objectives, real situational teaching content, care for different students, clear explanations, examples, fair and just teaching evaluations, time limits, and timely feedback, and adjustment of teaching influence effective teaching evaluation.
Effective communication → effective teaching evaluation	Teaching dialog between teachers and students and cooperative learning between teachers and students influence effective teaching evaluation.
Online learning → effective teaching evaluation	Video learning, homework learning, forum learning, learning time preference, and page access influence effective teaching evaluation.
Social support guarantee → effective teaching evaluation	Cognitive and emotional support from family, school, teachers, and classmates and support of the learning platform influence effective teaching evaluation.
Online course design → effective teaching evaluation	Course content, presentation mode, time flexibility of the course, richness and practicability of the content, flexibility of class hours, rich and exciting content, practicality and scientific selection of teaching materials, and reasonable layout of materials influence effective teaching evaluation.

### Theoretical saturation test

In this step of the study, 1/3 of the interview records were selected for the theoretical saturation test. The test results show that the categories in the model are richly developed, with no new important categories and relationships identified in addition to the six main categories that influence the effective teaching of MOOCs; in addition, no new components were found in the six main categories. Thus, the aforementioned “students’ MOOC learning behavior and effective teaching evaluation model” was deemed to have reached theoretical saturation.

## Results and discussion

**FIGURE 1:**
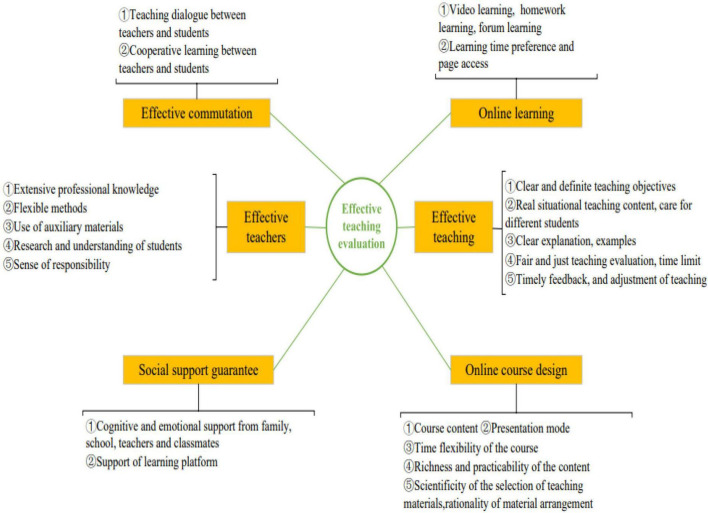
Students’ massively open online courses (MOOCs) learning behavior and effective teaching evaluation model.

The previous analyses reveal that the teaching evaluation in MOOCs can be effectively explained by the “students’ MOOCs learning behavior and effective teaching evaluation model” shown in Figure 1. Specifically, the factors influencing the effective teaching evaluation of MOOCs can be summarized into the following six main categories: effective teachers, effective teaching, effective communication, online learning behavior, social support guarantee, and online course design, all of which are consistent with the mechanisms of the effective MOOC teaching evaluation model (i.e., the ways and paths by which these factors influence effective teaching in MOOCs).

### Effective communication

Effective communication is central to the effective teaching of MOOCs. Teachers and students can communicate and talk via the online learning platform, exchange and answer questions in the platform’s discussion area, share ideas, and cooperate in learning. For example, using these platforms, students can participate in teachers’ scientific research projects and carry out cooperative research. The findings of the existing MOOC research show that students who often speak and communicate via the forum have a high graduation rate. Accordingly, it is highly important to encourage students to actively communicate with other course participants and teachers in the forum. Through sorting out our interview materials with students, we found that: An additional aspect of note is the importance of scientifically and effectively organizing and designing the meeting and discussion classes of MOOCs. This is consistent with the results of Peng and Xu (2020) that these aspects are an important means of assessing the students’ online learning and level of knowledge mastery, in the meantime, these can also help to improve relationships and communication between teachers and students, which play a pivotal role in the effective teaching of MOOCs. Accordingly, in MOOC meeting and discussion classes, key aspects such as epidemic prevention measures, pre-class organization, discussion topic design, on-site discussion, and teachers’ summaries should be carefully implemented.

### Effective teachers

Effective teachers represent the leading factors in effective teaching – the degree of participation, teaching level, organization and management level, and sense of responsibility of the leading teachers all have important effects on the teaching quality of MOOCs. In the last open question “Suggestions for Effective Teaching of this MOOC” in the questionnaire, more suggestions are targeted toward lecturers, such as “I hope the teachers’ voice can be vivid and humorous”, “the teachers’ teaching methods should be flexible,” and “I hope the teachers have a sense of responsibility and speak more about the ideological and political content of the course.” The lecturers’ teaching ability and accomplishments not only directly affect the delivery of teaching content but also influence the effects of the learning service itself. Additionally, if the course videos provided by MOOC lecturers display good language expression ability, inductive ability, content organization ability, and extensive professional knowledge, these factors will tend to have a positive impact on improving MOOC quality. Accordingly, lecturers involved in MOOC learning should take the initiative to study and understand their students, be aware of their students’ learning needs and difficulties, clarify the characteristics and expectations of online teaching in epidemic periods, and make efforts to rapidly master the required operational methods for online teaching design during an epidemic period. This is consistent with the results of Kim and Song (2022) that MOOC lecturers with full emotions, clear language expression, good image and temperament, humorous and interesting characteristics will be more attractive to students. According to our interview, we found an interesting conclusion that students in MOOCs during the epidemic period preferred the teachers to attend classes without masks, and they believed that teachers wearing masks hindered communication with each other. But in traditional classrooms, teachers are expected to wear masks and strictly observe quarantine measures.

### Online learning

Students’ online learning behavior patterns are strongly correlated with their learning outcomes. Some students’ online learning behaviors, such as homework completion ratio and video completion rate, have an important positive impact on their learning effect, whereas some online learning behaviors will negatively impact learning effects. Therefore, during MOOCs, guiding students to ensure positive online learning behaviors and avoid or minimize negative online learning behaviors is of vital importance in achieving optimal teaching effects. For example, considering group collaborative learning in MOOCs, course participants can be encouraged to choose to set up learning groups or temporarily set up online learning groups; these groups can be used to support learners through experiences such as case learning, collaborative design, practicing dividing tasks and collaborating in group activities, completing learning tasks together, and forming collaborative results (Admiraal et al., 2014). Secondly, MOOC learners should be trained to have a sense of learning responsibility-by cultivating this learning responsibility, the technical advantages of learning management systems can be applied to track the participation of MOOC learners and appropriate learning interventions can be provided as required to help them form habits such as autonomous learning and time management (Luo et al., 2014). For less active learners, sending “learning interventions” by email is relatively more effective; these participants tend to be less active due to procrastination or insufficient free time.

For example, we found in the survey of Guangzhou university MOOC teaching, MOOC lecturers rain (A new intelligent teaching management system jointly launched by Tsinghua University and XuetangX) has obtained the good teaching effect, students cannot easily get distracted and offline classes, because the rain classroom management system automatically records the student online learning behavior. Meanwhile, with rain class, teachers can push the pre-class preview courseware with MOOC videos, exercises and voice to students’ mobile phones, so that teachers and students can communicate and give feedback in time. Rain classroom can cover every teaching link of pre-class, in-class, and after-class, providing complete three-dimensional data support for teachers and students, personalized statements, automatic task reminder, so that teaching and learning are more clear. Teachers can assist MOOCs teaching through rain classroom, so as to have a real-time and more comprehensive understanding of students’ learning status and facilitate targeted teaching.

### Effective teaching

Effective teaching and effective practice complement one another. As an old saying goes, “sharp tools make good work.” Thus, teachers’ lesson preparation is particularly important, and the effort in advance directly determines the success or failure of specific operations. No matter how teaching is reformed, lesson preparation remains crucial, especially for teachers who lack MOOC teaching experience. Teachers should master the operating processes for MOOCs before classes to avoid low-level technical mistakes during lectures. In addition, they should be familiar with video materials and PPT courseware in MOOCs. Assignments also form an integral part of teaching. They are not only an effective means to consolidate and apply knowledge but also help to cultivate students’ ability to transfer their knowledge and work independently; in addition, assignments can provide timely and insightful feedback on teaching effects. To better consolidate teaching content, students are required to complete after-class exercises on MOOC platforms after each class and upload screenshots confirming the completion of the homework to the course QQ group or WeChat group for teachers to check and evaluate. When assigning homework, particular attention should be given to providing some enlightening and controversial questions rather than solely questions with a defined, standard answer, thus providing learners with a situation to which they can comprehensively apply the knowledge they have learned from the course to solve problems (Hone and Elsaid, 2016). For learners’ homework, the evaluation system built into MOOC platforms or peer evaluation can be used to provide course participants with feedback (Littenberg-Tobias and Reich, 2020).

### Online course design

Reasonable and effective classroom teaching design is key to improving the efficiency of network-based teaching of MOOCs. Teachers are the organizers and guides of classroom teaching in MOOCs. To achieve excellent online MOOC classroom teaching design, the course teacher must comprehensively consider aspects including effective communication with students on the network platform, sharing of knowledge, methods, and experience, exchanging feelings, experiences and ideas, promoting the effective implementation of network teaching, and ensuring positive interactions between course participants. MOOC teaching requires increased time and energy investment in the early stages of planning and process maintenance (Chen et al., 2017; Jung and Lee, 2018); in particular, the early stage of teaching design often requires teachers to invest 2–4 times as much time and energy as traditional curriculum teaching design, which plays a vital role in the final quality and effect of MOOC teaching (Hsu et al., 2018; Hollebrands and Lee, 2020). Both content design and teaching design should consider the course participants’ learning background, learning time, learning style, and learning ability, provide inclusive teaching content and engaging videos, arrange an appropriate number of tests with moderate difficulty, and allow learners to choose their own learning progress. MOOC teachers should ensure the following aspects are clear when designing their teaching: a detailed syllabus, the MOOC teaching themes, the MOOC teaching methods, and the knowledge and skills that will be acquired by learners. In addition, online teaching design during epidemic periods should include online teaching theory, online teaching mode, online teaching cases, online learning support services, etc. Online teaching modes during epidemic periods can be classified into the following four types: online live broadcast synchronous teaching mode, online course asynchronous teaching mode, online double-qualification cooperative teaching mode, and online mixed multi-element teaching mode.

### Social support guarantee

Effective teaching organization and management ensure effective teaching. Social support guarantees refer to respect, care, and attention from society during the process of online learning, including cognitive, emotional, and learning platform support from families, schools, teachers, and classmates, which have an important impact on students’ online learning motivation and effectiveness. With the emergence of numerous online teaching platforms, such as Rain Classroom, DingTalk, ZOOM Class, and Tencent Class, selecting the correct one is increasingly important because improper selection will negatively impact both teaching effect and quality. The biggest challenge involved in network teaching is not only to fully leverage the advantages of the rich resources and flexibility of network teaching platforms but also to avoid the disadvantages caused by poor interactions between teachers and students. A smooth and stable network environment is also a basic condition of online learning, ensuring that students can continue to conduct online learning effectively.

## Conclusion and policy recommendations

### Conclusion

In this paper, teachers’ and students’ MOOC teaching behavior was taken as the research object, and grounded theory was used to conduct semi-structured interviews with 30 university teachers and students who use MOOC teaching platforms. Based on existing research, the mechanisms influencing effective teaching in MOOCs were explored and explained in detail from the perspectives of students and teachers. An evaluation model of students’ MOOC learning behaviors and effective teaching was then constructed, which reveals and comprehensively analyzes the main factors that promote effective teaching in MOOCs, thus providing new insights into how to enrich the system of teaching of MOOCs. The study’s results show that effective teaching of MOOCs in an epidemic situation can be measured by constructing a multidimensional indicator system that considers (1) effective teaching, (2) effective teachers, (3) effective communication, (4) students’ online learning behaviors, (5) social support guarantees, and (6) online course design. The above six factors have important positive impacts on learning through MOOCs; among these factors, only certain online learning behaviors will negatively influence the effects of MOOC teaching and produce negative outcomes. Therefore, based on the findings in this paper, corresponding suggestions are proposed, such as attaching importance to group cooperative learning in MOOCs and cultivating MOOCs learners’ sense of learning responsibility. The results of this study have important theoretical implications for understanding the connotations of effective teaching of MOOCs, thus enriching and supplementing the theory of effective teaching and evaluation. These results can also provide practical guidance for teachers to effectively implement the teaching of MOOCs and promote the improvement of MOOC teaching quality. The theoretical contribution of this paper lies in exploring the key factors affecting MOOCS effective teaching during the epidemic period, and constructing a model of MOOC learning behavior and effective teaching evaluation, which provides a theoretical basis for teaching managers in universities to set up MOOC teaching evaluation index system. At the same time, this paper expands the application scope of grounded theory, especially in MOOC education research combined with the epidemic background.

### Policy implications

Based on the findings of this study, the following policy implications are proposed:

(1) Innovate teaching evaluation methods and set up a comprehensive evaluation index system combining “teacher teaching” and “student learning”. Existing MOOCs achieve their most effective teaching based on the teaching content, teaching methods, teaching attitude, and teaching effect (Xiao, 2017). These four elements can be used to evaluate courses, build an index system reflecting effective teaching behavior characteristics, and develop an assessment model of the main elements of teaching. As the primary participants in the classroom teaching process, students should also have the undisputed right to express whether they are satisfied with teachers’ teaching. MOOC teaching managers in colleges and universities should pay attention to the key role of students’ active learning in developing teaching quality, emphasize the influence of students’ learning characteristics, motivation, and self-management on teaching effects, and consider student course outcomes and subjective initiative, thereby helping to achieve students’ all-round development.

(2) Improve the evaluation index system and increase qualitative incentive evaluation. In addition, fully explore the learning evaluation function of MOOCs platform, improve the evaluation mode, emphasize dynamic process evaluation, and improve the degree of cooperation and enthusiasm of course teachers. For example, MOOC teaching managers in colleges and universities could consider setting up qualitative incentive evaluations, issuing questionnaires to identify and evaluate students’ opinions on educators’ teaching before they start teaching MOOCs, and encouraging students to provide positive feedback and praise to teachers as appropriate. At the end of the evaluation in this study, students were asked to conduct a qualitative incentive evaluation of the teacher’s MOOC teaching. First, the progress achieved by the teacher during the course was described, and then any shortcomings of the teacher’s MOOCs teaching were highlighted. These types of constructive, qualitative comments help to ensure that teachers feel that students are paying attention and are interested in their teaching. Through encouraging feedback, teachers can enhance their confidence and enthusiasm in teaching, improve the quality of MOOC teaching, and tailor their teaching style based on students’ comments.

### Research limitations and prospects

Due to time constraints, there are certain limitations in the research process in this work. For example, in this study, we only investigated students and teachers who are MOOC users and did not interview MOOC platform software developers. Therefore, the conclusions of this study are limited to analysis only from the perspective of MOOC teaching platform users. Accordingly, in future research, we plan to conduct field research on software developers to further verify and expand the study’s conclusions from multiple perspectives.

## Data availability statement

The original contributions presented in the study are included in the article/supplementary material, further inquiries can be directed to the corresponding author/s.

## Ethics statement

The studies involving human participants were reviewed and approved by Research Ethics Committee of Guangzhou University. Written informed consent for participation was not required for this study in accordance with the national legislation and the institutional requirements.

## Author contributions

JL and XW were contributed to conception of the study. JL was performed the formal analysis. XW was conducted the validation. YY wrote the first draft of the manuscript. All authors contributed to manuscript revision, read, and approved the submitted version.
